# Correction: A new extended gumbel distribution: Properties and application

**DOI:** 10.1371/journal.pone.0309768

**Published:** 2024-08-28

**Authors:** Aisha Fayomi, Sadaf Khan, Muhammad Hussain Tahir, Ali Algarni, Farrukh Jamal, Reman Abu-Shanab

The funding statement for this paper is incorrect. The correct Funding statement is as follows: This project was funded by the Deanship Scientific Research (DSR), King Abdulaziz University, Jeddah under the Grant No. number (KEP-PhD:21-130-1443). The authors, therefore, acknowledge with thanks DSR for technical and financial support.

There are errors in the Author Contributions. The correct contributions are:

Conceptualization: Sadaf Khan and Muhammad Hussain Tahir

Data curation: Ali Algarni

Formal analysis: Sadaf Khan and Reman Abu-Shanab

Investigation: Aisha Fayomi

Methodology: Reman Abu-Shanab

Project administration: Muhammad Hussain Tahir

Resources: Muhammad Hussain Tahir

Software: Aisha Fayomi

Supervision: Farrukh Jamal and Ali Algarni

Validation: Farrukh Jamal

Visualization: Sadaf Khan and Ali Algarni

Writing—original draft preparation: Sadaf Khan and Reman Abu-Shanab

Writing—review and editing: Sadaf Khan and Ali Algarni
