# Supplementary material for: A new extended gumbel distribution: Properties and application
Source: PLoS One. 2022 May 27;17(5):e0267142. doi: 10.1371/journal.pone.0267142 (PMC9140309; doi:10.1371/journal.pone.0267142)
Supplement: Attachment — Submitted filename: Response to Reviewers.pdf [file pone.0267142.s002.pdf]

Reviewer #1:

1. When claiming the start of limit of integers could be changed in formula (11), the author need to clarify why it could be verified by Mathematica, maybe list the result given by Mathematica, to make the point more convincing.

Reply: Thank you for pointing out; we stated this fact in order to maintain transparency. Further, due to the importance of the results defined in this section as all the further results in the manuscript are based on it. Kindly see the Mathematica results below:

$$\sum_{j=0}^{\infty} (-1)^{j} \,\mathrm{Binomial}[a, j]\, \mathrm{Exp}\left[-j\, (-\mathrm{Log}[G[x]])^{-1/\sigma}\right]$$

$$\left(1 - e^{-(-\mathrm{Log}[G[x]])^{-1/\sigma}}\right)^{a}$$

$$\sum_{j=1}^{\infty} (-1)^{j} \,\mathrm{Binomial}[a, j] \sum_{i=1}^{\infty} (-1)^{i} / i!\, j^{i}\, (-\mathrm{Log}[G[x]])^{-i/\sigma}$$

$$\left(1 - e^{-(-\mathrm{Log}[G[x]])^{-1/\sigma}}\right)^{a}$$

2. In section 3.6, there are 4 groups of parameter combinations but only 3 out of them are tested, the author should explain why they chose not to test all of them and explain how to decide which one to be tested.

Reply: Thank you for the comments. Now, all four of the parameter combinations are stated and tested in section 3.6. As far as the selection of the parameter combinations are concerned, we choose them randomly and included the results which converged most readily.

3. The author should make some explanation or do some analysis for figure 5 instead of just leaving it there.

Reply: Thanks for the suggestion; the cited issue has been taken care of.

4. At the beginning of section 3, the citation “The cdf and pdf…” should be Section 2.1.3 instead of 1.1.3.

Reply: Thanks for the correction; the cited issue has been implemented.

5. There are many typesetting errors and some punctuation errors, the author need carefully check the paper.

Reply: Thanks for the refinement; we have now improved the overall manuscript to the best of our abilities.

Reviewer #2:

<u>Major comments</u>

Major concerns relating to methods or significance is this work is very similar to

a) A. Ogunde et.al (https://www.hindawi.com/journals/jam/2020/2798327/)
b) Hormatollah Pourreza et.al (https://journals.sagepub.com/doi/full/10.1177/09622802211009262).

Did authors read these papers to compare the result?

Reply: With due respect to the reviewer, the cited papers are quite different to the proposed model. We explain this as follows

a) In the article, cited as A. A. Ogunde et.al (2020), *the authors proposed a special model of Gumbel- Type-II distribution* based on the generalization originally proposed by [1]Cordiero et al. (2013).

[1: Cordeiro, G. M., Ortega, E. M. M. & Cunha, D. C. C. (2013), 'The exponentiated generalized class of distributions', Journal of Data Science 11, 1–27.]

The cdf of Exponentiated Generalized Gumbel Type-Two (EGTT) distribution is given as

$$G(x) = \left[1 - \left(1 - e^{-\alpha x^{-v}}\right)^a\right]^b \qquad \therefore x \geq 0$$

$where\ a > 0, b > 0, \alpha > 0\ and\ v > 0.$

The EGTT model is right skewed with only one non-monotonic hazard rate shape (upside down bathtub). Two real life data sets have been analyzed with only two comparative models. However, the model presented in ***the newly proposed manuscript Equation 3***, given as follows

$$Z(x;\varphi) = 1 - \left(1 - e^{-e^{-x/\sigma}}\right)^{\theta} \qquad \therefore x \in \mathbb{R} \ and\ \sigma > 0, \theta > 0$$

Out of so many, the following are some of the significant points which shows the uniqueness of the proposed model, i.e.

i. The proposed model discusses the ***Gumbel-Type I*** distribution in the context of extreme value theory.
ii. The proposed model presents a ***generalization of Gumbel-Type I*** distribution, denoted by EGuG.
iii. The proposed model presents the properties of generalized class.
iv. The proposed model presents the functional forms of at least eleven special models, out of which the distribution function (cdf) and density functions (pdfs) of three models arising due to the cdf proposed in equation 3 are obtainable, which can be studied as a result of the proposed generalization.
v. Out of the three presented special models, Nadarajah Haghighi distribution is taken as baseline and the model EGuNH has further been studied thoroughly.
vi. The proposed special model outperforms ***five*** well established generalized classes of distribution along with the baseline model which solidifies its superiority over its existing generalizations.

b) In the article, cited as Hormatollah Pourreza et.al (2021), the authors proposed a generalization of two parameter Gamma distribution (***Gamma-X***) using the link function $W[G(x)] = -\log[1 - G(x)]$, following the methodology introduced by [2]Miroslav M. Ristic and Narayanaswamy Balakrishnan (2012) to propose the generalization of one parameter Gamma distribution and studied the Gamma exponentialted exponential (***GEE***) distribution. The cdf of Gamma-X family is given as

$$G(x) = \frac{\gamma(\alpha, -\beta ln\overline{F(x)})}{\Gamma\alpha} \qquad where\ \alpha > 0, \beta > 0\ and\ x \geq 0.$$

Further, the authors took Weibull as baseline and studied the properties of ***Gamma-Weibull*** distribution.

[2: Miroslav M. Ristić & Narayanaswamy Balakrishnan (2012): The gamma exponentiated exponential distribution, Journal of Statistical Computation and Simulation, 82:8, 1191-1206]

Regarding the usefulness and distinctiveness of the proposed model as compared to cited model, we present the following arguments

i. The proposed model presents a ***generalization of Gumbel-Type I*** distribution, denoted by EGuG, using the link function (also known as generator function) as

$$W[G(x)] = \log[-\log\{1 - G(x)\}]$$

ii. The proposed generalization is useful to ***unbounded interval*** of data with range $-\infty \leq x \leq +\infty$ which is unique only to this model.

iii. The proposed model discusses the ***Gumbel-Type I*** distribution in the context of extreme value theory with applications related to environmental and health hazards, an area which is under explored in the extreme value theory.

iv. Nadarajah haghighi distribution is used as baseline modelwhich is unique for zero inflated models unlike Weibull distribution.

v. The EGuNH model with only three parameters yields all four hazard rate shapes including monotone (Increasing & decreasing) and non-monotone (bathtub & upside down bathtub) whereas Gamma-Weibull model yields only three shapes of hazard rate function (Increasing, decreasing & Bathtub) with four parameters.

<u>Minor comments</u>

1. In the Abstract, the authors used the term “greatest likelihood method”. Is it the maximum likelihood method? If so, I think the common term should be the maximum likelihood method.

Reply: Thanks for pointing out; it was a typing error. It has been take care of.

2. In Section 1, the authors did not explain what T-X methodology is. It would be good if the authors can provide a citation.

Reply: Thank you for the valuable suggestion. T-X methodology is an integral part of modern distribution theory. As the article was already lengthy, we excluded it to keep the article length short and concise. However, we have now added a brief description and the citation of the T-X methodology paper in the manuscript.

3. In Section 2, it would be good to use the hazard rate function to define “hrf” rather than failure rate function.

Reply:

Thanks for pointing out; it has been dealt with as per suggestion.

4. In-Page 13, the authors did not cite figure # for “Plots of MGs for some parameter values”.

Reply:

Thanks for pointing out; it has been corrected.

5. The tables should be self-explanatory. Please explain the acronyms in the tables. For example, what is LoS in Table 12?

Reply:

Thanks for pointing out; it was a typing error. The acronym in the table has now been explained.

6. The authors seem did not explain most of figures and tables. For example, what is the message for Figure 13?

Reply: Risk measures are statistical indicators of the uncertainty of financial institutions or asset portfolios.

VaR is defined as the highest cash value estimated to be compromised over a specified time horizon at a certain confidence level. An alternate risk measure, expected shortfall, seeks to mitigate some of VAR's faults.

For example, if the 95 % one-month VaR is $1 million, there is a strong likelihood that the portfolio will not decline more than $1 million within next month.

These measures have been explained in section 3.3 and 3.4 of the manuscript in detail.

7. In the simulations, it would be good if the authors could report the coverage probability of the 95% confidence intervals as another evaluation metric for MLE estimation of model parameters.

Reply: Thanks for valuable comments; it has now been included as suggested.

8. In real data examples, the authors did not show how to interpret model parameters and their practical meanings in real data.

Reply: A parameter is a factor which influences the response or behavior of a mathematical entity yet is termed constant. In estimation section, the method of maximum likelihood estimation is used to estimate the model parameter mathematically. Given the significance of these parameter estimates, in application section, they are estimated numerically for each of the three real datasets.

Regarding practical meaning of model parameters, many risks are connected with each other, meaning that events in one system can trigger failures in another. For example, a tsunami can disrupt a country’s power transmission; a tsunami could disable the cooling and power supplies of the nuclear reactors causing a major environmental and human disaster.

In statistical quality control, the estimates of parameter are used to select which lot is likely to be included in the production process.

Reviewer #3:

I would have like to see how the extended Gumbel distribution behaves for global sequence alignment application. For example, Sardiu et al. 2005, showed that the score statistics of sequence alignment follows a Gumbel distribution. Is the

extended Gumbel distribution presented in this paper superior to the classical Tracy-Widom distribution for example for global alignments? I suggest the author cite this paper since the application of the Gumbel distribution in biological applications were used since 2005. The author does not talk in the manuscript about the Tracy-Widom distributions. This needs to be included in the introduction.

Reply: The cited issue has been addressed without loss of generality.

I would also suggest that the author comment more on the limitation of this distribution. How the distribution change when the size is large or too small, for example. Is it a critical point where the distribution does not apply anymore? These are important points which need to be addressed to prove superiority and utility of the extended Gumbel distribution.

Reply: In simulation section, we did comment on the empirical finding of the model parameters. The model parameters do not behave so well for small samples but as the sample size gets large, the mean squared error starts to decrease. Thus, suggesting a better fit for large sample case and rather acts inadequately for too small sample size.

Note: In revised manuscript, changes suggested by

- Reviewer 1 are in red
- Reviewer 2 are in blue
- Reviewer 3 are in green

It was the evident wish and will of the Reviewers that we make the changes to our manuscript on their insightful observations. Without losing the originality, we have tried to accommodate their suggestions in best possible manner. We have also improved the overall presentation of the paper.

We thank the Editor and the three referees for the constructive comments and hope that the revision is now appropriate for publishing.

Please, do not hesitate to contact me by e-mail if you have any questions. I look forward to hearing from you on this revised version.

Sincere Regards,
Sadaf Khan
(Corresponding author)
