# Supplementary material for: A new extended gumbel distribution: Properties and application
Source: PLoS One. 2022 May 27;17(5):e0267142. doi: 10.1371/journal.pone.0267142 (PMC9140309; doi:10.1371/journal.pone.0267142)
Supplement: Attachment — Submitted filename: Plos_review_2022.docx [file pone.0267142.s001.docx]

In the manuscript “A New Extended Gumbel distribution: Properties and Application”, Fayomi *et al.* introduced a new generalization of the Gumbel distribution. The author presented the distribution through the data simulation as well as three real-world applications.

The paper is very well presented, and the author provided a good review of the literature. The equations are well explained and clearly presented; I am glad to see that the author used the real-world applications to show the superiority of the distribution.

I would have like to see how the extended Gumbel distribution behaves for global sequence alignment application. For example, Sardiu et al. 2005, showed that the score statistics of sequence alignment follows a Gumbel distribution. Is the extended Gumbel distribution presented in this paper superior to the classical Tracy-Widom distribution for example for global alignments? I suggest the author cite this paper since the application of the Gumbel distribution in biological applications were used since 2005. The author does not talk in the manuscript about the Tracy-Widom distributions. This needs to be included in the introduction.

I would also suggest that the author comment more on the limitation of this distribution. How the distribution change when the size is large or too small, for example. Is it a critical point where the distribution does not apply anymore? These are important points which need to be addressed to prove superiority and utility of the extended Gumbel distribution.

Overall, the manuscript is good, however I suggest that the author address these concerns.
